# Controlling Plasmonic Catalysis via Strong Coupling with Electromagnetic Resonators

**DOI:** 10.1021/acs.nanolett.4c03153

**Published:** 2024-09-12

**Authors:** Jakub Fojt, Paul Erhart, Christian Schäfer

**Affiliations:** Department of Physics, Chalmers University of Technology, 412 96 Göteborg, Sweden

**Keywords:** Plasmonic Catalysis, Strong Light−Matter Coupling, Hot Carriers, Polaritonic Chemistry, Localized Surface Plasmon, Density-Functional Theory

## Abstract

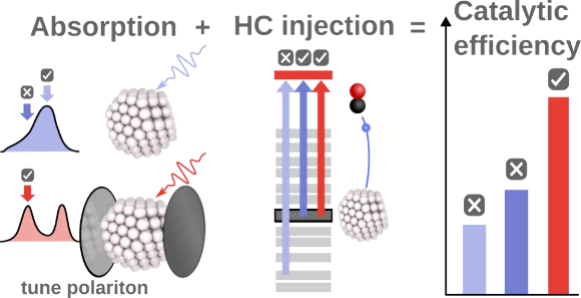

Plasmonic excitations decay within femtoseconds, leaving nonthermal (often referred to as “hot”) charge carriers behind that can be injected into molecular structures to trigger chemical reactions that are otherwise out of reach—a process known as plasmonic catalysis. In this Letter, we demonstrate that strong coupling between resonator structures and plasmonic nanoparticles can be used to control the spectral overlap between the plasmonic excitation energy and the charge injection energy into nearby molecules. Our atomistic description couples real-time density-functional theory self-consistently to an electromagnetic resonator structure via the radiation-reaction potential. Control over the resonator provides then an additional knob for nonintrusively enhancing plasmonic catalysis, here more than 6-fold, and dynamically reacting to deterioration of the catalyst—a new facet of modern catalysis.

Hot carrier (HC) technology, i.e., injecting HCs (nonthermal carriers) into a molecule^1^ or semiconductor,^2^ promises considerable improvements in light-harvesting,^2^ solar-to-chemical energy conversion,^3−5^ and catalysis.^6−11^ Commonly, HCs are generated in plasmonic nanoparticles (NPs) through the nonradiative decay of the localized surface plasmon (LSP), a mode of collective electronic motion that is excited by light. This process is highly efficient due to the large absorption cross section of plasmonic NPs at visible-near UV frequencies.^12−14^ One possible process of injecting those generated HCs into a molecule follows a *direct* HC transfer^15^ where charge-transfer excitations form with one carrier in the NP and the other in the orbital of a molecule. This “direct transfer/injection” is thus *not* sequential but directly creates HC in the molecule. We will use “HC injection” synonymously with this direct injection process in the following to stay consistent with the available literature. Such a direct process is more useful in terms of selectivity,^16^ has been experimentally observed^17−19^ and is at least as likely^15,19^ as the process of transferring HCs that are formed in the NP across the interface.^6^ The direct HC transfer process sensitively depends on the alignment of energetic levels comprising charge transfer and LSP excitations.^20^ Improving HC generation and injection are therefore critical to explore the full potential of plasmonic catalysis.

One possible angle to improve plasmonic catalysis is to control the interplay between plasmonic particles and an optical field. Confining optical modes, may it be via structured meta-surfaces or Fabry-Pérot cavities, results in an increase in interaction to a material with spectral overlap. From an increase in mode density follows, according to Fermi’s golden rule, an increase in photoabsorption cross section, which has been successfully employed to deposit more energy in plasmonic NPs, create more HCs, and thus further increase catalytic efficiency.^9,11,21−24^ At sufficiently strong interaction, light and matter hybridize into polaritonic quasi-particles that can enhance exciton^25−29^ or charge^30−33^ conductance and even control chemical reactivity.^34−41^ Plasmonic nanoparticle crystals^42,43^ can reach extreme light-matter coupling strength, entering the deep strong coupling domain, that even exceeds the excitation energy of the LSP of the NPs comprising the crystal.

In this Letter, we explore to which extent strong coupling between an optical resonator and a plasmonic Ag NP can be leveraged to control the catalytic effect on a nearby CO molecule. Our approach is based on energetic restructuring, i.e., each step in the catalytic process is associated with a set of energies and the efficiency of the full process is optimized by aligning those energies. In contrast to strongly coupling the molecule to the NP, which is suggested to lead to minor changes in HC transfer,^44^ our systems features strong coupling between the NP and the external resonator. Using an atomistic description of the NP-molecule system, we show that the microscopic mechanism of charge injection to the molecules is a dephasing of the LSP to charge-transfer excitations.^20,45,46^ We then couple the system to an optical cavity,^47^ giving rise to polaritonic states that emerge from hybridization of light and matter. The polaritonic states allow tuning of previously mismatched energies, providing nonintrusive control that increases the efficacy of HC injection into the molecule more than 6-fold. We conclude with a comprehensive discussion of potential applicability and limitations, including a comparative study for engineering the shape of the NP.

## System

**Figure 1:**
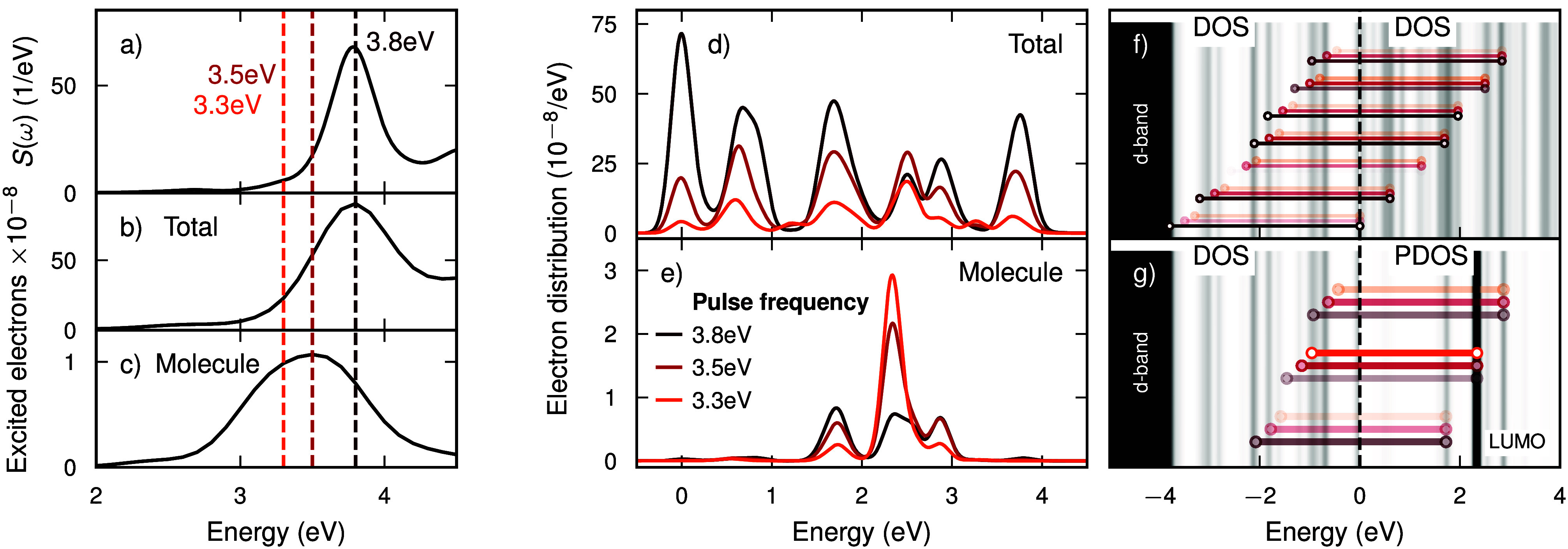
(a) Absorption spectrum of the NP-CO coupled system. The colors of the vertical dashed lines correspond to 3 exemplary excitation energies. (b) Number of excited electrons in the total system and (c) in the molecule. (d) Electron distribution in the total system and (e) projected on the molecule. (f) Schematic illustration of the energy-conserving excitations that contribute to excited electrons in total, overlaid on the total density of states (DOS). As there are many available states, there are many possibilities to satisfy the energy conservation condition (see text). (g) Schematic illustration of the energy-conserving excitations that contribute to excited electrons in the molecule, overlaid on the total DOS of unoccupied states and projected density of states (PDOS) of the molecule of unoccupied states. The sparse PDOS defines much stricter resonance conditions such that energetic matching becomes an essential component of catalytic efficiency. The opacity of the transitions in (f, g) is proportional to the relative probability of each particular excitation.

Our exemplary model system comprises a CO molecule near a 201-atom Ag NP (effectively 1.5 nm in diameter; Figure S1a). The LSP of the NP (resonance at 3.8 eV; see Figure 1a) and molecular excitations have no spectral overlap. Carriers that form on the molecule are then only due to a photocatalytic effect of the excited LSP dephasing into charge-transfer excitations—providing unambiguous insight into the HC injection. We place the molecule 3 Å from the (111) face of the NP, as the alignment is most clearly illustrated here, but discuss other distances in the SI. We excite the system with light polarized along the bond axis of the molecule. A detailed description of methodology and dephasing process can be found in the SI and refs (20 and 46).

## Hot-Carrier Transfer

A prerequisite for the generation of HCs is the absorption of energy. It is therefore not surprising that when varying the frequency of the driving pulse, the number of excited electrons in the system (i.e., NP and molecule) roughly follows the shape of the absorption spectrum (Figure 1a, b), as the amount of energy absorbed dictates how many carriers are excited. However, the number of hot electrons (HEs) injected to the molecule clearly deviates from this shape (Figure 1c) as HC transfer depends on generation and *injection* efficiency. The maximum injection is obtained at 3.5 eV, which is off-resonant to the LSP, and the number of injected HCs is roughly constant in a range of pulse frequencies between 3.3 and 3.6 eV. Thus, factoring out the efficiency of absorption (77% less energy is absorbed using, e.g., the 3.3 eV pulse compared to 3.8 eV; see Figure S2) the injection of HEs must be much more efficient using a pulse of 3.3 eV.^20^ In fact, by normalizing the number of injected carriers by the amount of energy absorbed at each pulse frequency (Figure 3; solid line), we construct a hypothetical injection efficiency that peaks at 3 eV. We return to discuss this later in the text. It should be pointed out that hot holes are also injected into the molecule, albeit to a much smaller extent (Figure S3), as the highest occupied molecular orbital (HOMO) is too far below the Fermi level given the pulse frequencies considered (Figure S4).

## Level Alignment

To understand why electron injection is more efficient off-resonance, let us give a perturbative perspective on HC generation. Driving the system with an external potential *V*_ext_ we perturb the density *δρ* and induce a potential *δV*. This is primarily the Coulomb potential of the LSP. HCs form as the LSP starts to decay,^46^ as their coupling to *V*_ext_ is much weaker than to *δV*. From Fermi’s golden rule we should expect a continuous drive of frequency ω to result in a rate of HE formation in state *a* to be 1/τ/ℏ^2^∑_*i*_(2 – *f*_*a*_)*f*_*i*_|*M*_*ia*_|^2^ · (1/[(ω – ω_*ia*_)^2^ + τ^–2^] + 1/[(ω + ω_*ia*_)^2^ + τ^–2^]) where ℏω_*ia*_ is the energy of excitation *i* → *a*, *f*_*i*_ and *f*_*a*_ occupation numbers (including spin degeneracy of 2) and τ a characteristic lifetime of carriers.^48^ The denominator represents the requirement for energy-conservation during the excitation event, i.e., the longer the lifetime, the sharper the resonant condition.^a^ The transition matrix element *M*_*ia*_ = ∫ d***r***(*V*_ext_ + *δV*[*δρ*])(*f*_*i*_ – *f*_*a*_)ψ_i_^*^(***r***)ψ_*a*_(***r***) includes excitations driven directly by the external field and excitations driven by the induced density. The latter is the only non-negligble term of *M*_*ia*_, and effectively represents the coupling strength of the LSP to each excitation times the coupling strength of the LSP to the driving field, through the dependence on *δρ*.

Mapping out all excitations in the system (Figure S5) we see a competition between two effects dictating the probability for an excitation to occur. The pulse should be aligned to (i) the excitation energy ω_*ia*_ in order to satisfy the resonance condition (∼1/(ω – ω_*ia*_)^2^) and (ii) the LSP resonance, as |*M*_*ia*_(ω)|^2^ scales with the amount of energy absorbed.

The total NP+molecule system involves many energy-conserving excitations due to the dense DOS of the NP (illustrated by the accordingly colored lines in Figure 1f), such that (i) plays a minor role and (ii) causes the HE distribution to rather uniformly decrease in amplitude when the pulse is detuned from the LSP (Figure 1d+b). However, only few energy-conserving *charge-transfer* excitations, i.e., from NP *to* molecule, exist (Figure 1g) due to the more discrete molecular level-structure. For our particular NP-molecule geometry, the lowest unoccupied molecular orbital (LUMO) is hybridized with the metal, forming three distinct states at 1.73, 2.33, and 2.87 eV above the Fermi level (Figure S4; note that there is also a less pronounced state at 2.53 eV). Across all pulse frequencies, an excitation from NP states at −0.93 eV to the middle orbital at 2.33 eV (ℏω_*ia*_ = 3.26 eV) contributes the most to electron transfer (Figure S5). The intensity of this excitation increases as the pulse frequency is tuned closer to 3.26 eV, thanks to the resonance condition, despite the absorption decreasing as the pulse is detuned from the LSP (3.8 eV). For a pulse frequency of 3.2 eV, this excitation makes up almost 40% of the electron transfer (almost 80% made up by just 10 excitations). In practice, computing a meaningful matrix element *M*_*ia*_ for this excitation is a nontrivial task, as HCs are formed continuously due to the time-dependent density due to the LSP. However, our current interpretation is that this excitation, and a few others, have particularily large matrix elements due to the overlap of the acceptor and donor wave functions with the potential from the LSP. An improved alignment to a few excitation energies (3.02, 3.26, and 3.54 eV among others; see Figure S5) as the pulse is red-detuned causes more HEs to be injected into the middle orbital 1.98 eV (Figure 1g). Competition with the alignment to the LSP (3.8 eV) as well as the presence of other charge-transfer excitations with different ω_*ia*_ ultimately causes the plateau in HE injection between 3.3 and 3.6 eV. Having control over the alignment criteria (i) and (ii) presents a clear way forward to optimize HC injection. We begin by exploring to which extent strong coupling to electromagnetic resonator structures can be used to tune polaritonic excitations and ultimately increase injection efficiency.

## Modifying the Resonance with Strong Coupling

**Figure 2:**
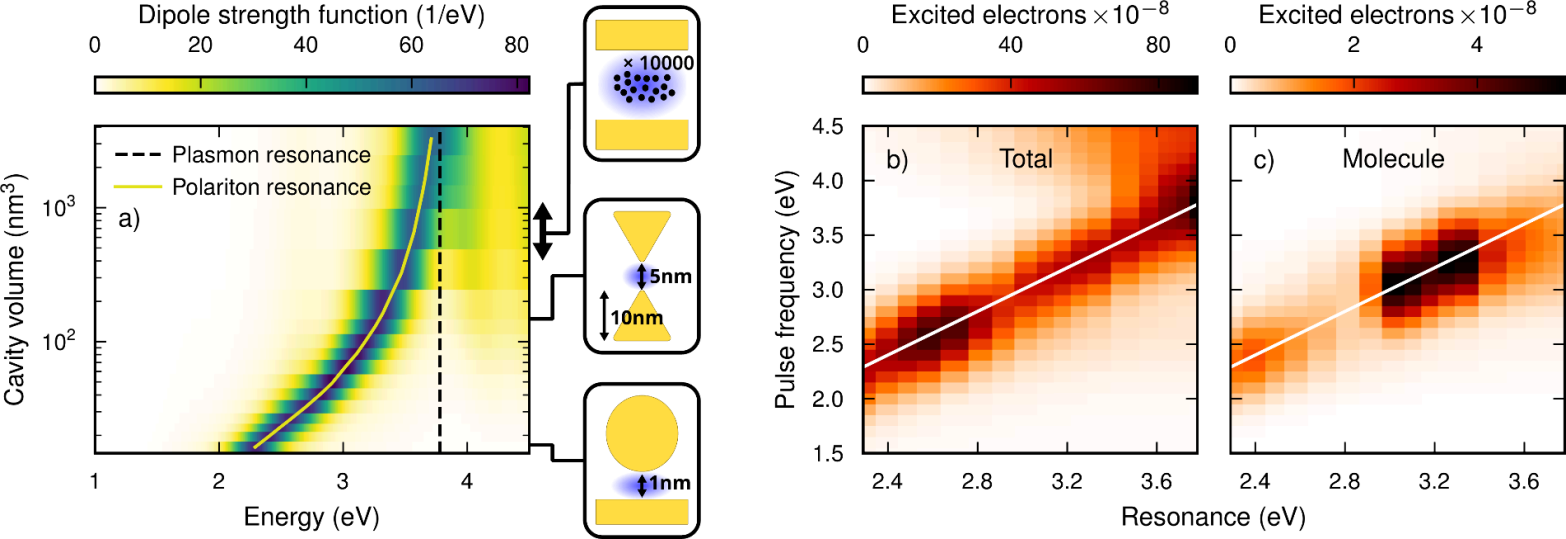
(a) Absorption spectrum of the NP-CO coupled system for different coupling strengths of the cavity. The spectral positions of the plasmon and the LP resonance are indicated by dashed and solid lines, respectively. The energetic position of the lower polariton (LP) resonance very closely follows the curve (3.805 eV – 6.2425 eVnm^3/2^/√*V*), where *V* is the cavity volume. The sketches mark effective cavity volumes that are possible to realize using (from the top) optical/meta-surface cavities and collective strong coupling (example assuming *N* = 10^4^, can be tuned by changing the density of emitters, further details in text), bow-tie antennas,^49^ and nanocavities.^50^ (b) Number of electrons excited in the system as a whole, (c) as well as in the molecule. The latter are plotted as a function of the spectral position of the resonance, which can be related to the cavity volume according to (a).

We introduce a simplified representation of a lossy cavity mode^b^ to our NP-molecule system via the radiation-reaction potential,^47^ as demonstrated in ref (51). The frequency of the cavity mode is tuned to the LSP resonance of 3.8 eV and the mode features a lifetime of τ = 17.32 fs, which is longer but still comparable to the lifetime of the LSP. Energy is absorbed from the pulse only via the matter system to ensure comparable results for all following investigations, i.e., increasing absorption efficiency is a secondary effect of resonator structures that we do not discuss in this manuscript but that has been explored in previous experiments. Here, we use an unspecified cavity structure to explore our hypothesis but illustrate possible realizations with typical effective cavity volumes in the insets of Figure 2a. In addition to a realization where a single NP-molecule system is strongly coupled to a small resonator, our discussion can be transferred to the collective coupling regime in which an ensemble of *N* NP-molecule subsystems couples collectively to e.g. an optical Fabry-Pérot cavity. In the collective limit, the HC injection efficiency of each NP-molecule subsystem will be smaller by a factor 1/*N*, but since an optical excitation is shared among *N* subsystems the total increase in catalytic efficiency of the entire system is identical to the simulations demonstrated in the following per absorbed photon (*N* × 1/*N*, linear response theory).^c^ The optical field follows Maxwell’s equations which leaves the electronic Kohn–Sham orbitals, and therefore condition (i), unaffected. Strong interaction between cavity and LSP causes the LSP to split in lower polariton (LP) and upper polariton (UP) (Figure 2a; the latter, however, is quenched at large coupling strengths due to overlapping with interband transitions). The clue is now that the LP can be monotonically red-shifted by reducing the effective mode volume of the resonator structure or increasing the density of optical emitters inside a given volume–we control condition (ii) by controlling the cavity.

Unsurprisingly, the total number of excited electrons in the system peaks when the system is driven in resonance with the LP (Figure 2b). Slightly shifting the LP approximately halves the number of electrons in the total system as the LSP is split into two polaritonic states that carry each half of the oscillator strength. Further increasing the cavity strength, the number of excited electrons stays roughly constant, with a notable exception between 2.5 and 2.8 eV. We attribute the increase in this region to a spectral overlap of the LP with a feature in the spectrum at 2.6 eV (Figure 1a; compare spectra of larger NPs in ref (46).).

**Figure 3:**
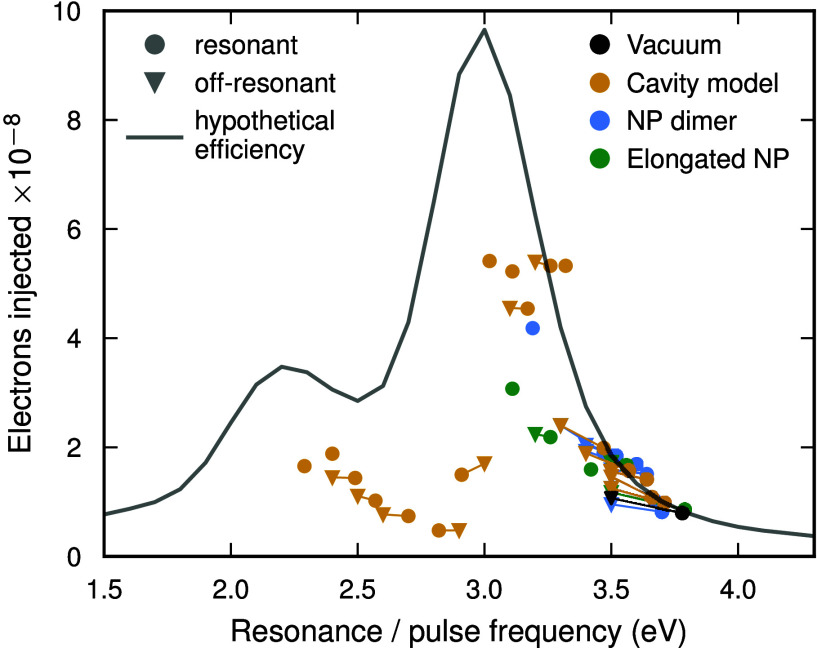
Number of electrons injected into the molecule for different setups. Because the principal charge-transfer excitation is red-detuned relative to the LSP resonance, transferring the most charge requires a trade-off between being tuned to the resonance (circles) and being tuned to the charge-transfer excitation. Imagining that we could “artificially” shift the resonance yields the solid gray line. The slight blue-shift of the in-cavity envelope and the artificially shifted resonance can be attributed to self-polarization effects.^47,52^ The data is normalized to correspond to the same absorption as the 201-atom vacuum system. In particular, the values for the NP dimer have been halved, and the values for the elongated NPs have been multiplied by 201 and divided by the number of atoms in each structure.

The number of electrons *injected* into the molecule does not necessarily peak at resonance, which we knew already from the no-cavity case. As illustrated in Figure 2, tuning the LP into the energetic domain of optimal injection efficiency at 3 eV results in a considerable increase of HEs injection. We increase the electron injection by a factor of 6.8 (the configurations with LP energy/pulse frequency of 3.02/3.0 eV, *V* = 63.2 nm^3^, and LP energy/pulse frequency of 3.26/3.2 eV, *V* = 131.2 nm^3^, tie for this record), compared to the cavity-free system excited at resonance (Figure 3; golden markers). Repeating this study for other distances between the NP and molecule results in similar, although weaker, behavior (Figure S7). In particular, at 2.2 Å distance, we obtain an increase by a factor of 2.6 using a LP energy/pulse frequency of 3.17/3.1 eV.

Strong coupling allows us to tune condition (ii) on demand and reach energetic alignment between optical absorption and HC injection. The ideal realization of a hypothetical system, in which the LSP can be freely tuned to match the pulse frequency, can be estimated by normalizing the injected carriers (at 3.8 eV pulse) by the amount of energy absorbed at each pulse frequency (Figure 3; solid line). The absolute maximum in HE injection for this hypothetical system would be then obtained when the pulse frequency, the LSP resonance, and the efficiency peak are all resonant. As established for our original system in vacuum (Figure 3; black), the real systems will not necessarily feature optimal injection for resonant (pulse vs LSP; circles) drive but perform typically best if driven slightly off-resonantly (triangles). Our proposed optical tuning mechanism via the LP follows the hypothetical efficiency indeed closely down to 3.2 eV. For even larger hybridization, a significant part of the energy is trapped in the cavity subsystem (Figure S6, Figure S7) and is no longer used to generate HCs which results in the under-performance below 3.2 eV.

## Atomistic Model Using NP Dimers

One possible atomistic realization of our resonator structure is to simply place a second identical NP close to the NP-molecule system (Figure S1b). LSPRs of the NPs hybridize, resulting in polaritonic eigenstates that resemble those of the generic cavity-NP system. The system is set up such that the gap of the NP dimer is between 9.83 Å (resonance 3.62 eV; see Figure S8) and 4.33 Å (resonance 3.35 eV) and the molecule is placed on the outer side of one of the NPs, so that charge can only be injected from that NP. The driving field is polarized along the axis connecting the NP dimer and CO molecule (*z*-axis). Normalizing by the increased amount of energy absorbed in this system, the number of injected electrons for this NP dimer system (Figure 3; blue markers) follows again the hypothetical efficiency.

## Manipulation of Shape

Popular strategies to control the optical properties of NP systems include the manipulation of shape, size, and composition.^53−57^ However, such approaches modify the electronic ground state which results in an overall change of optical and catalytic activity.^58^ We construct a series of artificially elongated NPs (Figure 3; green markers) by inserting up to 8 atomic layers in the middle of the structure and relaxing the nuclear positions with a simple effective medium theory model (Figure S1c–g; more details in SI). This procedure yields structures with aspect ratios from 1 to 2.14 and ensures that the tail of the NP, where the molecule is positioned, remains a (111) surface in order to be directly comparable to our other results, however the structures differ from conventional nanorods that can be fabricated. The simplistic relaxation results in a minor shift of the LSP injection efficiency even for the 201 Ag NP. Elongating the NPs, and normalizing the injection efficiency by the increase in size, leads to a mild increase in catalytic activity. Changing the shape modifies the ground state and with it the single-particle spectrum, moving the previously observed resonance out of reach. Shape manipulation is clearly a valid alternative but our study illustrates that both approaches feature unique strengths and a holistic optimization strategy that accounts for shape, size, composition, and polaritonic control holds great potential to give birth to a new generation of catalytic materials.

## Conclusions and Outlook

To summarize, we have illustrated that the efficiency of the direct HC transfer process depends on the alignment of the incoming photon energy, the LSP resonance of the NP, and the excitation energy of a few (or even a single) charge-transfer excitations.^20^ We have discussed a polaritonic framework for tuning the NP resonance to the charge-transfer excitations based on strong coupling to an electromagnetic resonator structure. In this framework, we achieve a more than 6-fold increase in HC injection with the potential to fine-tune this increase nonintrusively by adjusting the configuration of the resonator. Alternative strategies, based on modifying the NP shape, change LSP and charge-transfer excitations at the same time, resulting in a more complex optimization/control problem. The basic objective of all such techniques remains the same—reaching energetic alignment between optical absorption and injection, thus finding an optimum on the “hypothetical efficiency” curve introduced in Figure 3. Explicitly simulating NP dimers, one possible realization of NP-resonator hybridization, follows indeed closely our simplified cavity model as well as the hypothetical efficiency curve. Notable upsides for the use of electromagnetic resonator structures over the adjustment of shape or composition are 3-fold: (i) A chosen catalyst can be fine-tuned to a specific orbital and thus reaction, allowing for a more modular design-approach. (ii) Some resonator structures can be used to dynamically adjust to changes of the catalyst, appearing, e.g., due to deterioration. (iii) The established approach to boost absorption characteristics, and thus photocatalytic activity, with optical resonators^9,11,21−24^ can be conveniently combined with our approach.

Our proposal could be validated in various mixed-plasmonic or collectively coupled systems, many of which are already in striking distance^9,43,59,60^ or even exceed^10,42,60^ the necessary coupling strength. Minor remaining challenges are the design of resonator structures that have a sufficient quality factor and ensuring that the energy of the field is deposited into the photocatalytic NPs. A promising strategy could be the combination of photocatalytic NPs and dynamically switchable metasurfaces^61^ with machine learning supported free-form design.^62^ Such a system would be a possible candidate to reach appreciable enantiomer selective catalysis by using a crossover between chiral polaritonics and plasmonics.^63,64^ Maxwell-TDDFT approaches, especially those following embedding concepts,^47,51^ are ideal to support this task as they provide a computationally accessible framework to consistently link macroscopic optical energy distribution to microscopic HC dynamics. Recall, that direct HC transfer stands in competition with indirect transfer and catalytic increase due to heating^1,65−68^—a competition that is sensitive to various characteristics of the material and selection rules imposed by symmetry of its surface.^69,70^ Understanding the delicate HC injection process with the help of the adjustable nonintrusive control-knob discussed here would deepen our understanding of plasmonic catalysis and open new avenues for refined designs that will be required in the near future to elevate the hydrogen economy to the desired level. This includes the connection between energy absorption and chemical reactivity^71,72^ as well as the impact of prolonged nonequilibrium carrier distribution (before thermalization to HCs) on the reactivity.

To this end, polaritonically steered plasmonic catalysis might open a path to replace precious materials, such as platinum and gold, with more abundant and optically active materials, such as aluminum,^73^ thus triggering the next development step toward green chemistry.

## Data Availability

The data generated in this study are openly available via Zenodo at https://zenodo.org/records/13357330.

## Supporting Information Available

Atomic structures, computational details, energy absorbed by the no-cavity system, time-resolved number of hot carriers in total and in the molecule, (projected) density of states, transition contribution map, energetic contributions matter and cavity subsystems, electron injection for different NP-molecule distances, and absorption spectra (PDF)

## Footnotes

a We note that in real-time time-dependent density functional theory (RT-TDDFT) with an adiabatic exchange correlation (XC)-kernel, decay channels such as Auger and phonon scattering are missing and the lifetime is infinite. Since we are simulating a finite pulse with a full-width at half-maximum of 0.7 eV in frequency space, the resonant condition is broadened accordingly.

b We use the simplified expression  (in atomic units). Here, **ϵ**_*z*_ is the polarization of the cavity, *V* is the effective mode-volume, ω_*c*_ is the cavity frequency (set in resonance to the LSPR), and τ is the lifetime of the optical mode which is connected to the quality factor of the cavity.

c If the polaritonic states overlap with a broad darkstate distribution, a fraction of the energy deposited in the polaritons will leak into the dark states. That fraction (energy lost to dark states/energy provided) would follow the standard injection probabilities which lowers the fraction of our enhancement.
