# Supplementary material for: Controlling Plasmonic Catalysis via Strong Coupling with Electromagnetic Resonators
Source: Nano Lett. 2024 Sep 12;24(38):11913–20. doi: 10.1021/acs.nanolett.4c03153 (PMC11440648; doi:10.1021/acs.nanolett.4c03153)
Supplement: Supplementary file 1 — nl4c03153_si_001.pdf [file nl4c03153_si_001.pdf]

Supplementary Information: Controlling Plasmonic Catalysis via Strong Coupling with Electromagnetic Resonators

Jakub Fojt,[1] Paul Erhart,[1] and Christian Schäfer[1,*]

[1] *Department of Physics, Chalmers University of Technology, 412 96 Göteborg, Sweden*

(Dated: September 10, 2024)

CONTENTS

Supplementary Figures 2
S1. Atomic structures 2
S2. Amount of energy in the no-cavity system 2
S3. Hot-carrier generation over time 3
S4. Density of states and projected density of states of the molecule 4
S5. Transition contribution map for the 3 Å distance, no cavity case 5
S6. Amount of energy in the electronic and cavity subsystems 6
S7. Number of electrons injected for all considered systems 7
S8. Absorption spectra 8

Supplementary Methods 9
Atomic structures 9
Computational details 9

10

* Electronic address: christian.schaefer.physics@gmail.com

SUPPLEMENTARY FIGURES

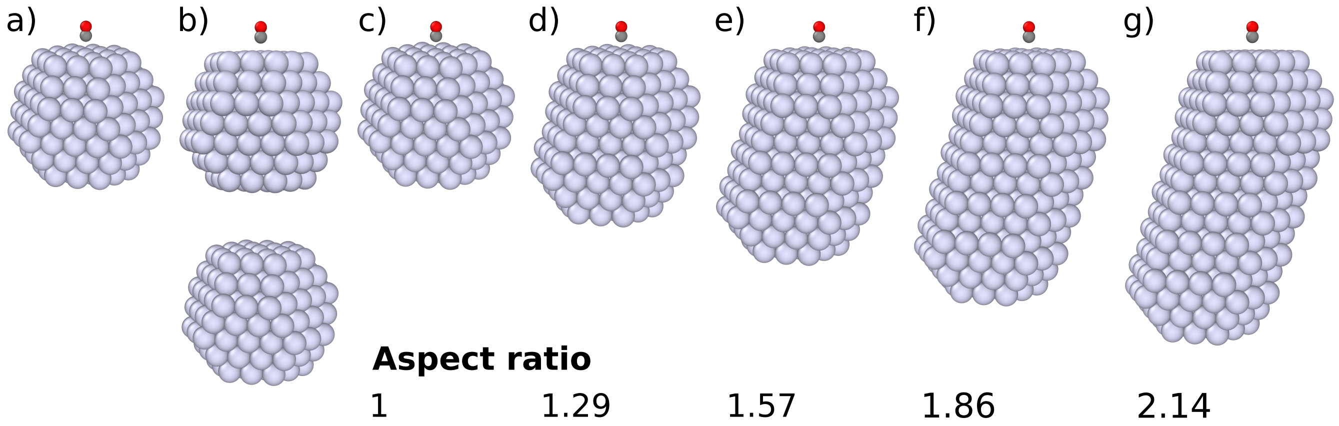

Figure S1. **Atomic structures.** (a) The regular-truncated-octahedron shaped NP with one CO molecule. (b) The NP dimer with the CO molecule placed near one of the NPs. (c-g) A series of elongated NPs with the CO molecule. The elongated structures have been constructed in such a way that the top and bottom (from the point of view of the figure) of the structures are {111} surfaces. The placement of the CO molecule is identical in all structures (a)-(g), centered on the (111) face with its bond axis perpendicular to the surface. In the RT-TDDFT calculations the polarization of the applied field is directed along the bond axis.

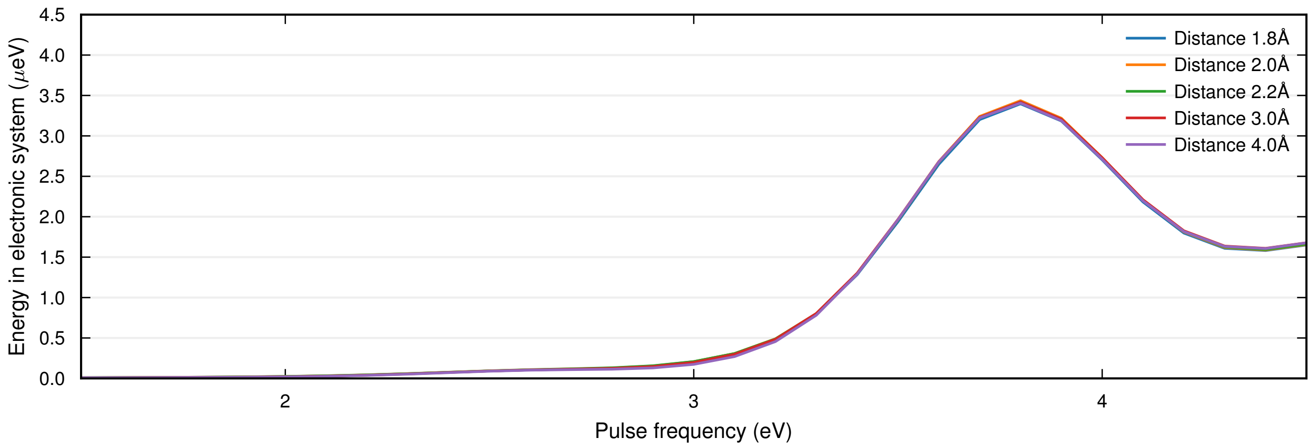

Figure S2. **Amount of energy in the no-cavity system.** Evaluated at the end of the simulation and plotted by pulse frequency. The position of the CO molecule barely makes a difference to the amount of energy absorbed in total.

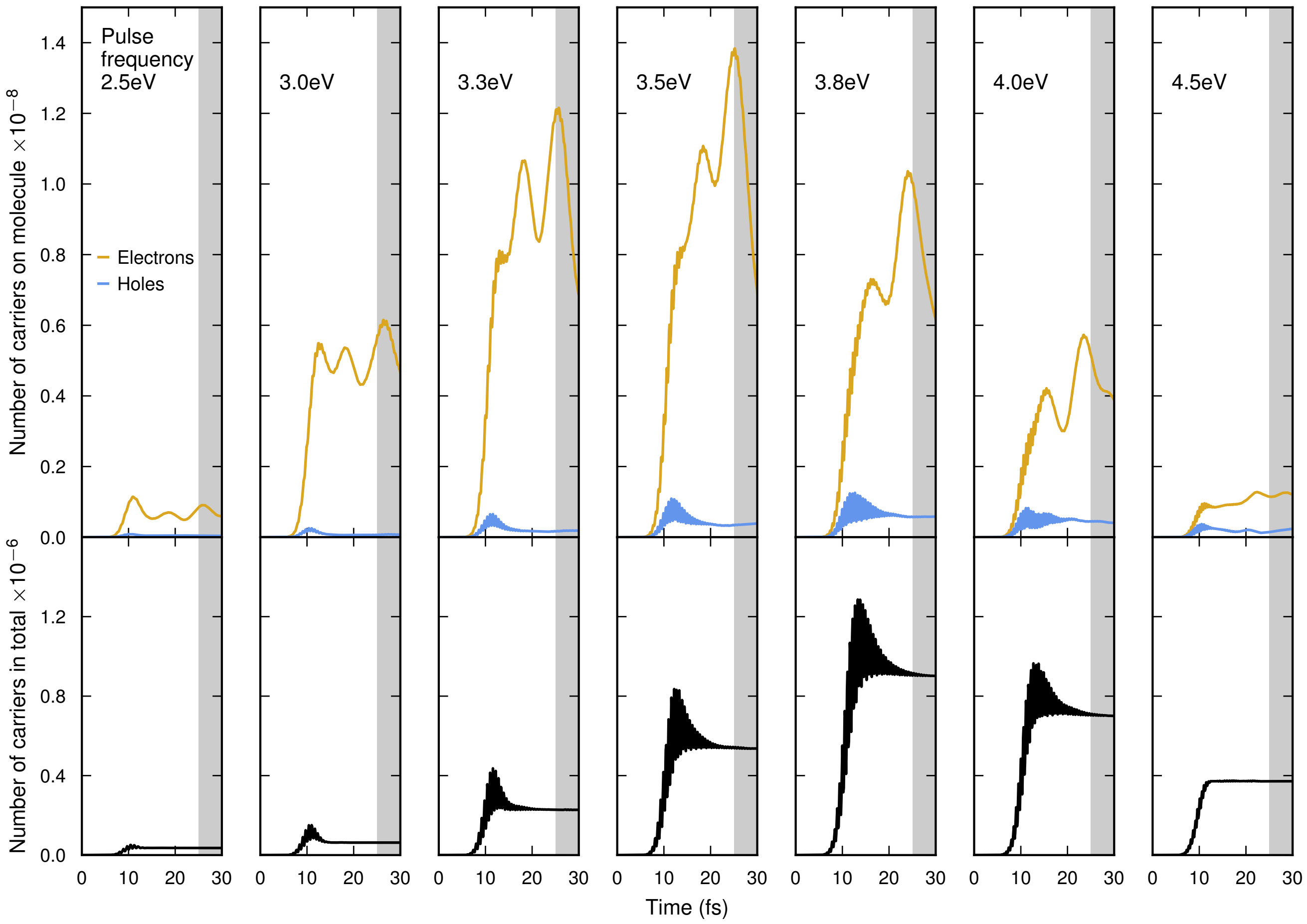

Figure S3. **Hot-carrier generation over time.** For the $3\,\text{Å}$ distance no-cavity system. The number of carriers increases as the plasmon dephases around the $10\,\text{fs}$ mark. While the total number of carriers reaches a steady value towards the end of the simulation, the number of electrons and holes in the molecule fluctuates. At any given time the number of electrons in the molecule is roughly a factor 10-100 smaller than the total number of carriers, and the number of holes in the molecule is a roughly a factor 5-10 smaller than the number of electrons in the molecule. The grey areas mark the time window where an average number of carriers was computed.

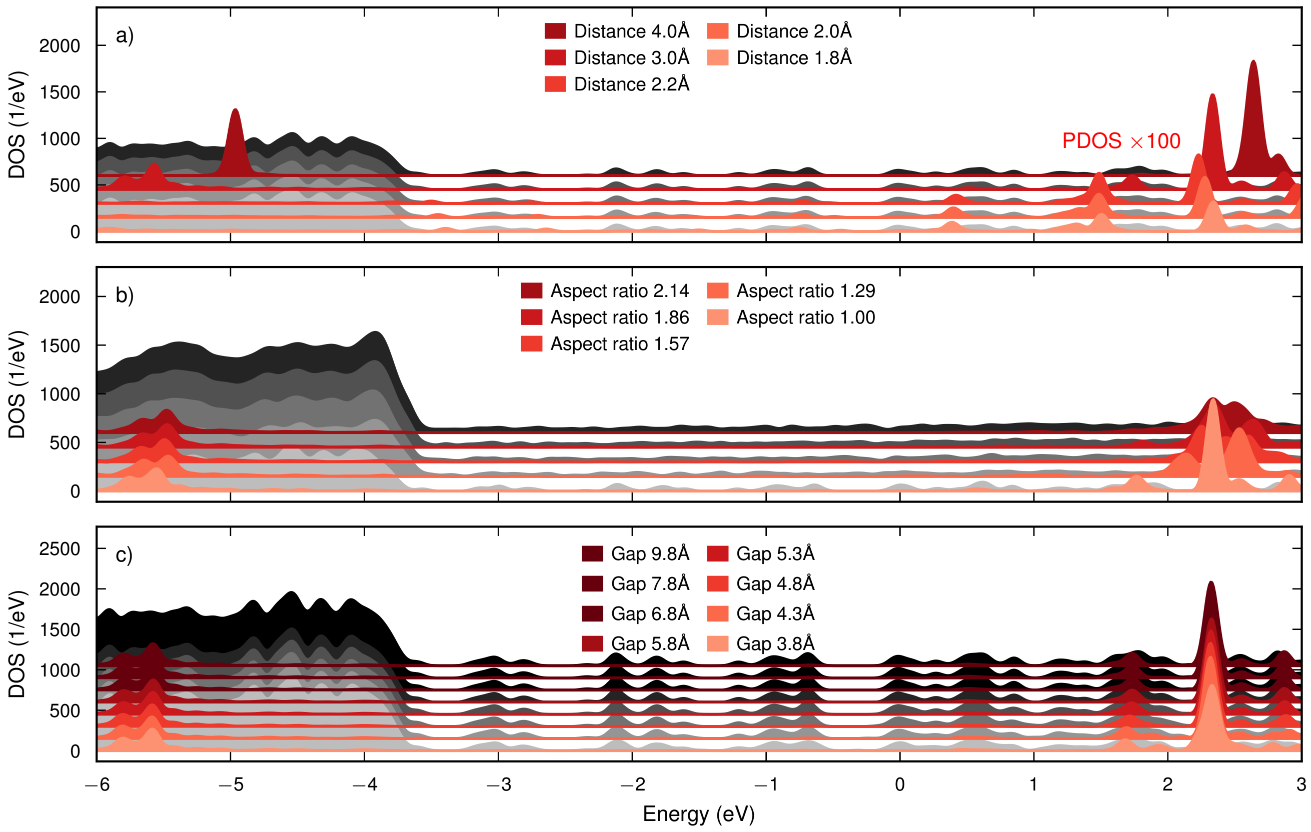

Figure S4. **Density of states and projected density of states of the molecule.** (a) For the NP-molecule system at various distances, (b) for the distance of $3\,\text{Å}$ between the molecule and elongated NPs, and (c) for the two NP, one molecule systems. For distances closer than $3\,\text{Å}$ the molecule is strongly hybridized with the NP, splitting the original LUMO state into many. Elongating the NP by adding several atomic layers to it also affects the hybridization, despite the molecule being kept at the same distance from the surface. Varying the gap in the NP dimer keeps the ground state unchanged, except for at the very smallest gaps, where tiny shifts in the DOS and PDOS are visible. Because the HOMO state is $5\,\text{eV}$ below the Fermi level at large distances, hole transfer to the molecule is virtually impossible. At smaller distances, hybridized branches of the HOMO appear closer to the Fermi level making hole transfer possible, but less prevalent than electron transfer.

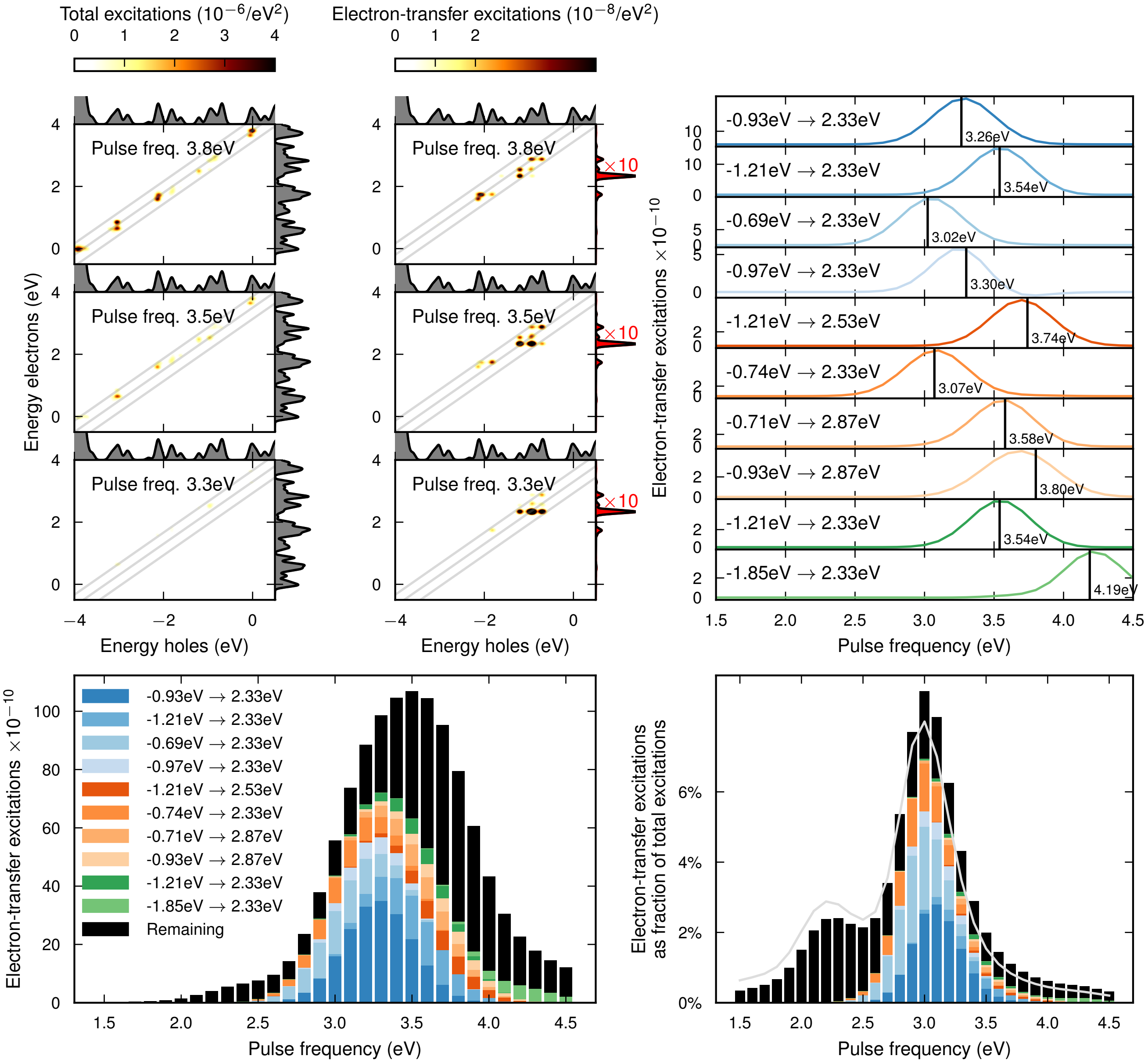

Figure S5. **Transition contribution map for the $3\,\text{Å}$ distance, no cavity case.**
(Top left) The contribution map to total excitations (NP and molecule), as well as projected on the molecule are shown after excitation with three different pulse frequencies.
(Top right) The 10 strongest electron-transfer excitations (that is contribution to the projected transition contribution map) for all pulses between $1.5\,\text{eV}$ and $4.5\,\text{eV}$ are shown. Transitions between degenerate states have been summed and plotted together. The strongest transition is one from the $-0.93\,\text{eV}$ NP state to the $2.33\,\text{eV}$ molecular state, which contributes the most to the number of electrons transferred when the pulse frequency is resonant with the transition energy of $3.26\,\text{eV}$.
(Bottom left) Alternative visualization of the 10 strongest excitations in the top right panel. For each pulse frequency, the amount of each excitation is stacked in color-coded bars, in front of a black bar that represents the total number of electron-transfer excitations (i.e. the data in Fig. 3 in the main text). This shows that at a pulse frequency of $3.2\,\text{eV}$, the $-0.93 \to 2.33\,\text{eV}$ excitation makes up $40\,\%$ all electron-transfer excitations. The 10 strongest excitations add up to almost $80\,\%$ of all electron-transfer excitations. At larger pulse frequencies, those excitations contribute less to electron-transfer because they are further away from their alignment (NP-DOS to LUMO) criterion.
(Bottom right) Same as bottom left, but the values have been divided by the number of HCs generated in total, showing that electron-transfer excitations make up about $7\,\%$ of all HCs when using a pulse frequency of $3\,\text{eV}$. This quantity is closely related to the "hypothetical efficiency" of figure 3 in the main text (which is number of electrons transferred divided by amount of energy absorbed; plotted as a grey line).

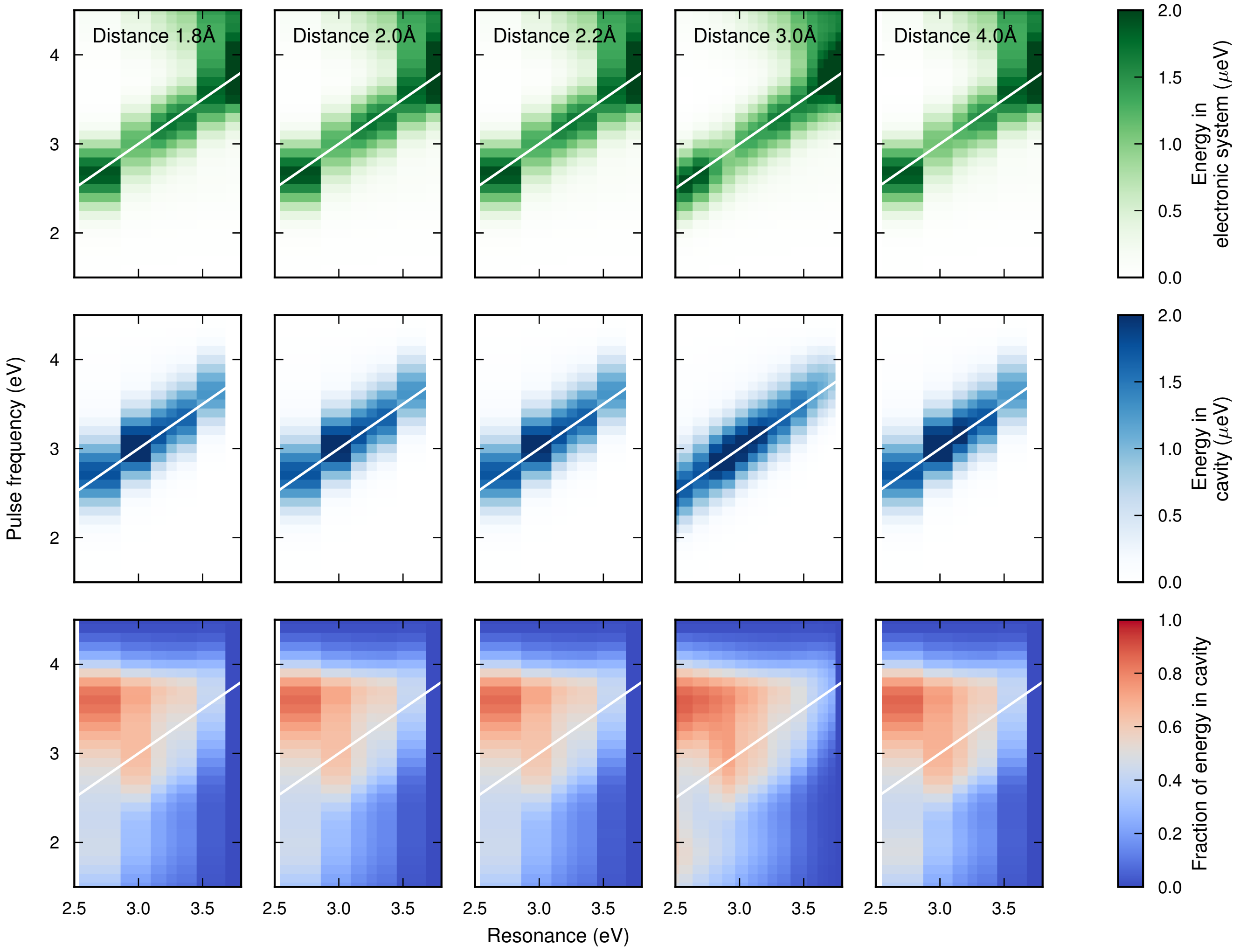

Figure S6. **Amount of energy in the electronic and cavity subsystems.** The energy supplied by the pulse is at the end of the simulation partially in the electronic subsystem and partially in the cavity subsystem. In the case that the resonance is redshifted away from the bare cavity resonance at $3.8\,\mathrm{eV}$, pulses resonant with the bare cavity tend to put a larger fraction into the cavity. Pulses above the d-band edge tend to put a larger fraction of energy into the electronic system.

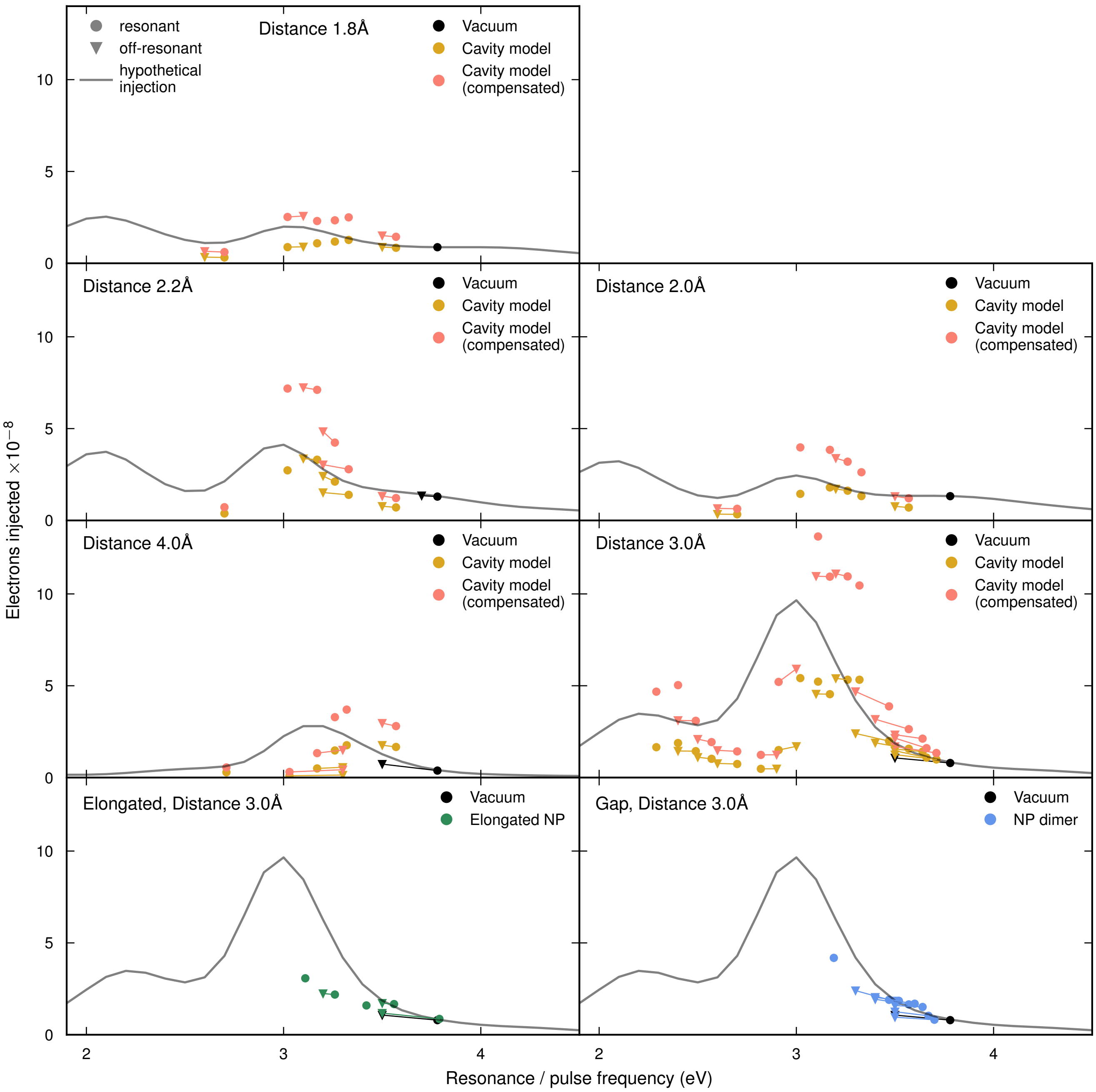

Figure S7. **Number of electrons injected for all considered systems.** Collection of results for different NP-molecule distances, elongated NPs, and NP dimers presented consistent with Fig. 3 of the main manuscript. Elongated and NP dimer efficiencies are normalized such that the same amount of energy is absorbed (see main manuscript) to ensure a fair comparison. As detailed in Fig. S6 and mentioned in the main manuscript, the cavity acts as as energy deposit such that only a fraction of the absorbed energy can be used to generate HCs. Compensating for this 'lost' energy by scaling the efficiency by the energy missing in the matter system ("Cavity model (compensated)"; orange) pushes the efficiency even beyond the hypothetical curve. This suggests that the LSP changes its character when strongly coupled to the cavity, emphasizing the need for reliable theoretical investigations that go beyond optimization based on the simplistic hypothetical curve. Very small distances between the NP dimers results in strong hybridization which in turn strongly modifies the LSP (see Fig. S8) and leads to the outlier at 2.6 eV resonance energy.

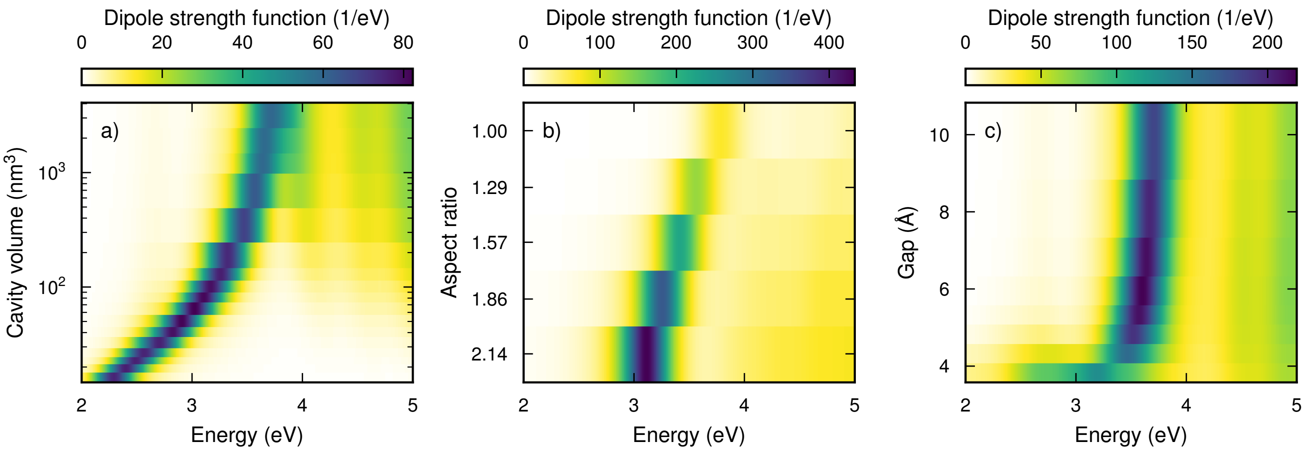

Figure S8. **Absorption spectra.** (a) For the $3\,\text{Å}$ distance system in an optical cavity. The coupling strength to the cavity is inversely proportional to the cavity volume. (b) For the NPs which have been elongated by inserting atomic layers in the middle. As the number of atomic layers is added, the number of electrons in the system, and thus total absorption increases. (c) For the NP dimer. For the smallest gap, the spectrum is drastically broadened.

SUPPLEMENTARY METHODS

Atomic structures

The atomic structure for the 201-atom Ag NP was taken from from Ref. 1. This NP has the shape of a regular truncated octahedron. In Ref. 1, the NP was relaxed with VASP [2–4] using a plane-wave basis set, the PAW [5, 6] method, the vdW-df-cx [7–10] XC-functional, a plane wave cutoff 500 eV, and Gaussian occupation number smearing scheme with the parameter 0.1 eV. Structure relaxations were performed using the conjugate gradient relaxation method implemented in VASP. Relaxation was stopped when the maximal force on any atom fell below 0.015 eV Å^{-1}.

For the analysis in the main text, we place a CO molecule with the bond length 1.144 Å at a distance of 3 Å from the center atom of the (111) face of the NP. The molecule is oriented with the C atom towards the surface, and the bond axis perpendicular to the surface (Fig. S1a). The polarization of the applied field in the TDDFT simulation is chosen along the bond axis. After constructing the NP+molecule structure, it *is not* relaxed further, in order to study the charge transfer at a fixed distance. In the SI, we also present results using structures where the distances are 1.8, 2.0, 2.4 and 4 Å.

For the analysis in the main text, we construct a NP dimer by duplicating the NP and shifting it a distance along the bond axis of the molecule. The CO molecule is added at a distance of 3 Å in the same configuration as before, in such a way that it is on the outer side of the NP dimer (Fig. S1b). We shift the NP so the center-center distance is between 24 and 18.5 Å, corresponding to an edge-to-edge distance of between 9.83 and 4.33 Å. In the SI, we also present an additional data point where the center-center distance is 18 Å and the edge-to-edge distance 3.83 Å.

For the analysis in the main text, we construct a series of artificially elongated NPs, by splitting the NP in the middle, shifting the structures apart to make room for two new layers, and duplicating the two atomic layers in the middle of the NP and placing them in the gap. To preserve the surfaces of the NP, the shifted half also has to be shifted laterally, in order to create a tilted structure. This is repeated between 0 and 4 times to create structures with between 0 and 8 added layers, corresponding to aspect ratios of between 1 and 2.14 (Fig. S1c-g). Here, we define the aspect ratio to be the ratio between the smallest number of atomic layers between two opposing {111} faces (which is always 7) and the largest number of atomic layers between two opposing {111} faces (which is 7 plus the number of added layers). These structures are relaxed with the EMT method and BFGS optimizer implemented in ASE [11] until the maximal force is 0.001 eV Å^{-1}.

Computational details

We carried out DFT and real-time TDDFT calculations using the GPAW package [12, 13] with LCAO basis sets [14], LCAO-RTTDDFT implementation [15], and the ASE library [11].

For the computation of the ground state we used the GLLB-SC [16, 17] XC-functional, utilizing the Libxc [18] library. We used the *pvalence* [15] basis set for Ag, which is optimized to represent bound unoccupied states, and the dzp basis set for CO. The structures were padded with at least 6 Å of vacuum, and we used a grid spacing of 0.2 Å for wave functions and 0.1 Å for potentials. The Coulomb potential was represented in numerical form on the grid, with an additional analytic moment correction [19] centered at the NP. Fermi-Dirac occupation number smearing with width 0.05 eV was used. The self-consistent loop was stopped when the integral of the difference between two subsequent densities was less than 1×10^{-12}. Pulay [20]-mixing was used to accelerate the ground state convergence.

We applied a δ-kick [21] of strength 10^{-5} in atomic units, and performed time propagation in steps of 10 as for a total length of 30 fs using the adiabatic GLLB-SC kernel. The Fourier transform of the Kohn-Sham density matrix was built up on the fly during propagation and saved on a predefined frequency grid for later analysis. The optical cavity was included in the simulations using the radiation-reaction potential [22, 23], implemented in a publicly available branch of GPAW [24].

For the computation of HCs, including projections on the molecule, we used the method of Refs. 1, 25, which is implemented in the rhodent package [26]. Details are found in the references. In short, we post process the saved Fourier transform the the KS density matrices to calculate the response to a Gaussian pulse

$$\mathcal{E}_z(t) = \mathcal{E}_0 \cos(\omega_0 t) \exp(-(t - t_0)^2/\tau_0^2) \tag{S1}$$

of frequency ω_0, strength $\mathcal{E}_0 = 51\,\mu\text{V}\,\text{Å}^{-1}$, peak time $t_0 = 10\,\text{fs}$, and duration $\tau_0 = 2.1\,\text{fs}$ (corresponding to a FWHM in frequency space of 0.7 eV). Because the number of electrons injected to the molecule does not reach a steady state, we present values that are averages in the time window between 25 and 30 fs in the simulation (see Fig. S3).

We computed the total density of states as

$$\sum_k \delta(\varepsilon - \varepsilon_k) \tag{S2}$$

and the PDOS for the molecule as

$$\sum_k \delta(\varepsilon - \varepsilon_k) \int_{\text{mol}} \left| \phi_k^{(0)}(\boldsymbol{r}) \right|^2 \mathrm{d}\boldsymbol{r}, \tag{S3}$$

where ε_k and $\phi_k^{(0)}(\boldsymbol{r})$ are the KS eigenvalues and wave functions, and the integral goes over the Voronoi region of the molecule (that is each point in space which is closer

to the molecule than any other atom). For visualization, the δ-functions in energy were replaced by a Gaussian $(2\pi\sigma^2)^{-1/2}\exp(-\varepsilon^2/2\sigma^2)$ with width $\sigma = 0.07\,\text{eV}$.

---

[1] J. Fojt, T. P. Rossi, M. Kuisma, and P. Erhart, Hot-Carrier Transfer across a Nanoparticle–Molecule Junction: The Importance of Orbital Hybridization and Level Alignment, Nano Lett. **22**, 8786 (2022).

[2] G. Kresse and J. Hafner, Ab initio molecular dynamics for liquid metals, Phys. Rev. B **47**, 558 (1993).

[3] G. Kresse and J. Furthmüller, Efficient iterative schemes for ab initio total-energy calculations using a plane-wave basis set, Phys. Rev. B **54**, 11169 (1996).

[4] G. Kresse and J. Furthmüller, Efficiency of ab-initio total energy calculations for metals and semiconductors using a plane-wave basis set, Nato. Sc. S. Ss. Iii. C. S. **6**, 15 (1996).

[5] P. E. Blöchl, Projector augmented-wave method, Phys. Rev. B **50**, 17953 (1994).

[6] G. Kresse and D. Joubert, From ultrasoft pseudopotentials to the projector augmented-wave method, Phys. Rev. B **59**, 1758 (1999).

[7] M. Dion, H. Rydberg, E. Schröder, D. C. Langreth, and B. I. Lundqvist, Van der Waals Density Functional for General Geometries, Phys. Rev. Lett. **92**, 246401 (2004).

[8] K. Berland and P. Hyldgaard, Exchange functional that tests the robustness of the plasmon description of the van der Waals density functional, Phys. Rev. B **89**, 035412 (2014).

[9] J. Klimeš, D. R. Bowler, and A. Michaelides, Chemical accuracy for the van der Waals density functional, J. Phys. Condens. Matter **22**, 022201 (2009).

[10] G. Román-Pérez and J. M. Soler, Efficient Implementation of a van der Waals Density Functional: Application to Double-Wall Carbon Nanotubes, Phys. Rev. Lett. **103**, 096102 (2009).

[11] A. H. Larsen, J. J. Mortensen, J. Blomqvist, I. E. Castelli, R. Christensen, M. Dułak, J. Friis, M. N. Groves, B. Hammer, C. Hargus, E. D. Hermes, P. C. Jennings, P. B. Jensen, J. Kermode, J. R. Kitchin, E. L. Kolsbjerg, J. Kubal, K. Kaasbjerg, S. Lysgaard, J. B. Maronsson, T. Maxson, T. Olsen, L. Pastewka, A. Peterson, C. Rostgaard, J. Schiøtz, O. Schütt, M. Strange, K. S. Thygesen, T. Vegge, L. Vilhelmsen, M. Walter, Z. Zeng, and K. W. Jacobsen, The atomic simulation environment—a Python library for working with atoms, J. Phys. Condens. Matter **29**, 273002 (2017).

[12] J. J. Mortensen, L. B. Hansen, and K. W. Jacobsen, Real-space grid implementation of the projector augmented wave method, Phys. Rev. B **71**, 035109 (2005).

[13] J. J. Mortensen, A. H. Larsen, M. Kuisma, A. V. Ivanov, A. Taghizadeh, A. Peterson, A. Haldar, A. O. Dohn, C. Schäfer, E. Ö. Jónsson, E. D. Hermes, F. A. Nilsson, G. Kastlunger, G. Levi, H. Jónsson, H. Häkkinen, J. Fojt, J. Kangsabanik, J. Sødequist, J. Lehtomäki, J. Heske, J. Enkovaara, K. T. Winther, M. Dulak, M. M. Melander, M. Ovesen, M. Louhivuori, M. Walter, M. Gjerding, O. Lopez-Acevedo, P. Erhart, R. Warmbier, R. Würdemann, S. Kaappa, S. Latini, T. M. Boland, T. Bligaard, T. Skovhus, T. Susi, T. Maxson, T. Rossi, X. Chen, Y. L. A. Schmerwitz, J. Schiøtz, T. Olsen, K. W. Jacobsen, and K. S. Thygesen, GPAW: An open Python package for electronic structure calculations, J. Chem. Phys. **160**, 092503 (2024).

[14] A. H. Larsen, M. Vanin, J. J. Mortensen, K. S. Thygesen, and K. W. Jacobsen, Localized atomic basis set in the projector augmented wave method, Phys. Rev. B **80**, 195112 (2009).

[15] M. Kuisma, A. Sakko, T. P. Rossi, A. H. Larsen, J. Enkovaara, L. Lehtovaara, and T. T. Rantala, Localized surface plasmon resonance in silver nanoparticles: Atomistic first-principles time-dependent density-functional theory calculations, Phys. Rev. B **91**, 115431 (2015).

[16] O. Gritsenko, R. van Leeuwen, E. van Lenthe, and E. J. Baerends, Self-consistent approximation to the Kohn-Sham exchange potential, Phys. Rev. A **51**, 1944 (1995).

[17] M. Kuisma, J. Ojanen, J. Enkovaara, and T. T. Rantala, Kohn-Sham potential with discontinuity for band gap materials, Phys. Rev. B **82**, 115106 (2010).

[18] S. Lehtola, C. Steigemann, M. J. T. Oliveira, and M. A. L. Marques, Recent developments in libxc — A comprehensive library of functionals for density functional theory, SoftwareX **7**, 1 (2018).

[19] A. Castro, A. Rubio, and M. J. Stott, Solution of Poisson's equation for finite systems using plane-wave methods, Can. J. Phys. **81**, 1151 (2003).

[20] P. Pulay, Convergence acceleration of iterative sequences. the case of scf iteration, Chem. Phys. Lett. **73**, 393 (1980).

[21] K. Yabana and G. F. Bertsch, Time-dependent local-density approximation in real time, Phys. Rev. B **54**, 4484 (1996).

[22] C. Schäfer and G. Johansson, Shortcut to self-consistent light-matter interaction and realistic spectra from first principles, Phys. Rev. Lett. **128**, 156402 (2022).

[23] C. Schäfer, Polaritonic chemistry from first principles via embedding radiation reaction, The Journal of Physical Chemistry Letters **13**, 6905 (2022), pMID: 35866694, https://doi.org/10.1021/acs.jpclett.2c01169.

[24] Public GPAW fork `https://gitlab.com/christian.schaefer.physics/gpaw/-/tree/qed/`. Please note that the implementation is experimental and the API is subject to change.

[25] T. P. Rossi, P. Erhart, and M. Kuisma, Hot-Carrier Generation in Plasmonic Nanoparticles: The Importance of Atomic Structure, Acs Nano **14**, 9963 (2020).

[26] J. Fojt, T. Rossi, and P. Erhart, rhodent – Analyzing hot-carrier distributions from TD-DFT simulations (2024), accessed: 2024-09-07.
